# Prospective Multicenter Trial Assessing the Impact of Positive Peritoneal Cytology Conversion on Oncological Outcome in Patients with Endometrial Cancer Undergoing Minimally Invasive Surgery with the use of an Intrauterine Manipulator: Positive Peritoneal Cytology Conversion and Its Association with Oncological Outcome in Endometrial Cancer

**DOI:** 10.1245/s10434-022-12356-9

**Published:** 2022-09-04

**Authors:** Franziska Siegenthaler, Silke Johann, Sara Imboden, Nicolas Samartzis, Haiyan Ledermann-Liu, Dimitri Sarlos, Markus Eberhard, Michael D. Mueller

**Affiliations:** 1 Department of Obstetrics and Gynecology, Bern University Hospital and University of Bern, Bern, Switzerland; 2 Department of Obstetrics and Gynecology Spitalzentrum Oberwallis, Standort Visp, Visp, Switzerland; 3 Department of Gynecology and Obstetrics, Canton Hospital Schaffhausen, Schaffhausen, Switzerland; 4 Department of Obstetrics and Gynecology, Canton Hospital Aarau, Aarau, Switzerland

## Abstract

**Background:**

Minimally invasive surgery is the standard approach in early-stage endometrial cancer according to evidence showing no compromise in oncological outcomes, but lower morbidity compared with open surgery. However, there are limited data available on the oncological safety of the use of intrauterine manipulators in endometrial cancer.

**Patients and Methods:**

This prospective multicenter study included patients with endometrial cancer undergoing laparoscopic staging surgery with the use of an intrauterine manipulator. We obtained three different sets of peritoneal washings: at the beginning of the surgical procedure, after the insertion of the intrauterine manipulator, and after the closure of the vaginal vault. The rate of positive peritoneal cytology conversion and its association with oncological outcomes was assessed.

**Results:**

A total of 124 patients were included. Peritoneal cytology was negative in 98 (group 1) and positive in 26 (group 2) patients. In group 2, 16 patients presented with positive cytology at the beginning of the surgery (group 2a) and 10 patients had positive cytology conversion during the procedure (group 2b). Recurrence rate was significantly different among the study groups, amounting to 9.2%, 25.0%, and 60.0% for groups 1, 2a, and 2b, respectively (*p* < 0.001). Group 1 showed the best recurrence-free and overall survival, followed by group 2a, while patients in group 2b had the worst oncological outcomes (*p* = 0.002 and *p* = 0.053, respectively). Peritoneal cytology was an independent predictor of recurrence and death on multivariable analysis.

**Conclusion:**

A total of 8.1% of patients with endometrial cancer undergoing minimally invasive surgery with intrauterine manipulation showed positive peritoneal cytology conversion associated with significantly worse oncological outcome.

Endometrial cancer is the most common gynecological tumor in developed countries and has a generally favorable prognosis with an overall 5-year survival rate of 80%.^1^ Its primary treatment consists of surgery including total hysterectomy, bilateral salpingo-oophorectomy, and nodal staging, if indicated.^2–4^ Nowadays, minimally invasive surgery is the standard approach in early-stage endometrial cancer according to evidence of prospective randomized trials showing no compromise in oncological outcomes but lower morbidity and shorter hospital stay compared with open surgery.^2,5–12^

In minimal invasive hysterectomy, the use of intrauterine manipulators is well established as it facilitates uterine mobilization, improves surgical field exposure, and provides a landmark for the colpotomy. Despite this, detailed information regarding its safety in oncological patients remains scarce, and clinical evidence substantiating the assumed mechanism of prevention of ureter injuries has not been found.^13^ Current studies on the effect of intrauterine manipulation during minimally invasive surgery on oncological outcomes in patients with endometrial cancer show contradicting results.^14–19^ However, the largest trial, on 2661 patients, revealed an association of the use of intrauterine manipulators with higher risk of recurrence, lower disease-free survival, and higher risk of death in endometrial cancer.^20^ The hypotheses generated are iatrogenic weakening of the myometrium through the manipulator leading to macroscopic uterine rupture and opening of the tumor into the peritoneal cavity or tumor spill via microscopic dissemination secondary to the increased pressure generated by the uterine device.^20–22^

Microscopic peritoneal metastasis and spreading outside the uterine cavity are suspected when cytopathological examination of pelvic washings demonstrates malignant cells. Positive peritoneal cytology is highly predictive of survival in multiple gynecological malignancies.^23^ In early-stage endometrial cancer, the prognostic importance of positive peritoneal cytology has long been debated,^24^ and in 2009, the Federation International de Gynecologie et Obstetrique (FIGO) removed cytology as a staging criteria from the endometrial cancer staging system.^25^ Nonetheless, multiple studies have proven the association of positive peritoneal cytology with decreased survival in patients with endometrial cancer.^26–30^ Furthermore, treatment of patients with positive cytology with adjuvant chemotherapy was associated with increased survival.^26^

There is evidence that intrauterine manipulation may result in retrograde seeding of the peritoneal cavity, as Sonoda et al. found higher incidence of positive peritoneal cytology in patients undergoing minimally invasive hysterectomy with the use of an uterine manipulator compared with open surgery.^31^ Nevertheless, these findings have not been confirmed in subsequent studies.^11,12,27^ Moreover, it has not been investigated to date whether positive cytology conversion caused by uterine manipulation has an effect on oncological outcomes in endometrial cancer.

With our study, we aim to analyze the association of intrauterine manipulation, peritoneal cytology, and oncological outcomes in patients with endometrial cancer.

## Patients and Methods

This is a prospective, multicenter, single-arm study. The experimental protocol was approved by the local ethics commission (reference number: 061/09) and meets the guidelines of the responsible governmental agency. All patients signed informed consent. Patients were included at the following three different centers in Switzerland: Department of Obstetrics and Gynecology at the Bern University Hospital, the Canton Hospital Schaffhausen, and the Canton Hospital Aarau.

The primary objective of the study was to assess the rate of positive peritoneal cytology conversion defined as an initially negative peritoneal cytology becoming positive during the course of the surgery. Secondary endpoints were recurrence rate, pattern of recurrence, time to first recurrence, and survival in relation to the peritoneal cytology. Patients were included if they were 18 years or older, were diagnosed with histologically confirmed and apparently early-stage endometrial cancer in preoperative workup, were willing to undergo minimally invasive staging procedure, and consented to follow-up. Exclusion criteria were the following: preoperative evidence of extrauterine disease including bulky lymph nodes, relapsed endometrial cancer at time of inclusion, presence of medical conditions contraindicating general anesthesia or standard laparoscopic surgery, concurrent diagnosis of ovarian cancer, and conversion to laparotomy. Routine preoperative imaging included transvaginal ultrasound and chest X-ray. Additional workup consisting of a thoracic, abdominal, and pelvic computed tomography (CT) scan was performed in patients with high-grade or non-endometrioid histology and/or suspicion of extrauterine disease.

### Study Intervention

All surgeries were performed using laparoscopy. Patients were positioned in the dorsal lithotomy position with both legs supported in stirrups. After achieving CO_2_ pneumoperitoneum, the whole abdominal cavity was visualized. Patients were tilted in the Trendelenburg position, and the first pelvic irrigation was conducted using 200 ml of lactated Ringer’s solution and sent for cytological evaluation (premanipulator washing). Bipolar coagulation of both fallopian tubes was performed before the insertion of the intrauterine manipulator. According to the surgeon’s choice, either the RUMI (CooperSurgical, Trumbull, CT) or Hohl (KARL STORZ AG, Tuttlingen, Germany) intrauterine manipulator was used. A second pelvic washing was obtained using 200 ml of lactated Ringer’s solution after the insertion of the manipulator and sent for cytologic evaluation (postmanipulator washing). Laparoscopic hysterectomy and bilateral salpingo-oophorectomy were then performed. The specimen was removed throughout the vagina and sent for frozen section evaluation. After closure of the vaginal vault, the third peritoneal cytology was collected with 200 ml of lactated Ringer’s solution (posthysterectomy washing). The indication for pelvic and paraaortic lymph node dissection was based on frozen section evaluation of the uterus according to international guidelines.^1,37^ All procedures were performed by surgeons with extensive experience in minimally invasive surgery. The three different sets of peritoneal washings were reviewed by board-certified cytopathologists to determine the presence or absence of malignant cells. In case of indeterminate results on conventional hematoxylin–eosin staining, immunohistochemistry on pan-cytokeratin and Ber-EP4 was conducted.^38,39^ According to the results of the peritoneal cytology, we formed the following groups for further analysis: group 1, with negative peritoneal cytology, and group 2, with positive peritoneal cytology. Group 2 was further subdivided into group 2a, with positive premanipulator washing, and group 2b, with a negative premanipulator but positive postmanipulator and/or posthysterectomy washing. Adjuvant treatment was carried out according to national and international guidelines.^1,37^

### Data Collection

Patient demographic, surgical, and histological data were collected prospectively in a central database. Demographic variables included age, body mass index (BMI), menopausal status, parity, previous history of tubal sterilization, and preoperative workup with hysteroscopy. Surgical variables included type of uterine manipulator, surgical staging procedure, and occurrence of uterine perforation. From final pathology, we gathered peritoneal cytology, histological subtype, FIGO stage (2009), presence of lymphovascular space invasion (LVSI), and tumor grading. We further collected data on adjuvant treatment, time of follow-up, recurrences, pattern of relapse, and vital status. Data on ethnicity have not been prospectively investigated.

### Outcomes

All patients received follow-up examination according to international guidelines.^1,37^ Time to first recurrence was defined as time elapsed from primary staging surgery to first recurrence. Recurrence-free survival was defined as time from primary staging surgery to first recurrence or death of any cause. Overall survival was defined as time from primary staging surgery to death of any cause. Patients who were alive were censored at the date of their last follow-up. Recurrences were classified into locoregional, abdominal, and distant recurrences according to the first site of recurrence. Locoregional recurrences included vaginal and pelvic recurrences (including pelvic lymph nodes and local spread to rectum and bladder); abdominal recurrences refer to recurrences outside the pelvis and are consisting of peritoneal carcinomatosis, omental metastasis, and paraaortic lymph node involvement; distant recurrences entail lung, liver, bone, and brain metastases as well as lymph node involvement other than pelvic and paraaortic. Simultaneous locoregional and abdominal recurrence was considered abdominal recurrence; simultaneous abdominal and distant recurrence was considered distant recurrence; and simultaneous locoregional and distant recurrence was considered distant recurrence.

### Sample Size and Statistical Analysis

Assuming a rate of positive peritoneal cytology conversion of 6% during the course of surgery, with an alpha error of 0.05 and a statistical power of 85%, the estimated sample size was 120 patients. Statistical calculations were performed using the Statistical Package for Social Sciences (IBM SPSS Statistic version 25.0). Categorical variables were reported as frequencies and percentages, while continuous variables were reported as means and standard deviations. Patients, tumor, and treatment characteristics were analyzed using chi-square statistics or Fisher's exact test in case of categorical and *t*-test or analysis of variance (ANOVA) for continuous variables. Survival curves were generated using the Kaplan–Meier method and compared using the log-rank test. Univariable Cox regression analyses were conducted to assess the relationship between the risk of recurrence and death with other prognostic factors. Any variables significant on univariable analysis were included in the multivariable analysis after testing for collinearity. A *p*-value of less than 0.05 was considered statistically significant.

## Results

**Fig. 1:**
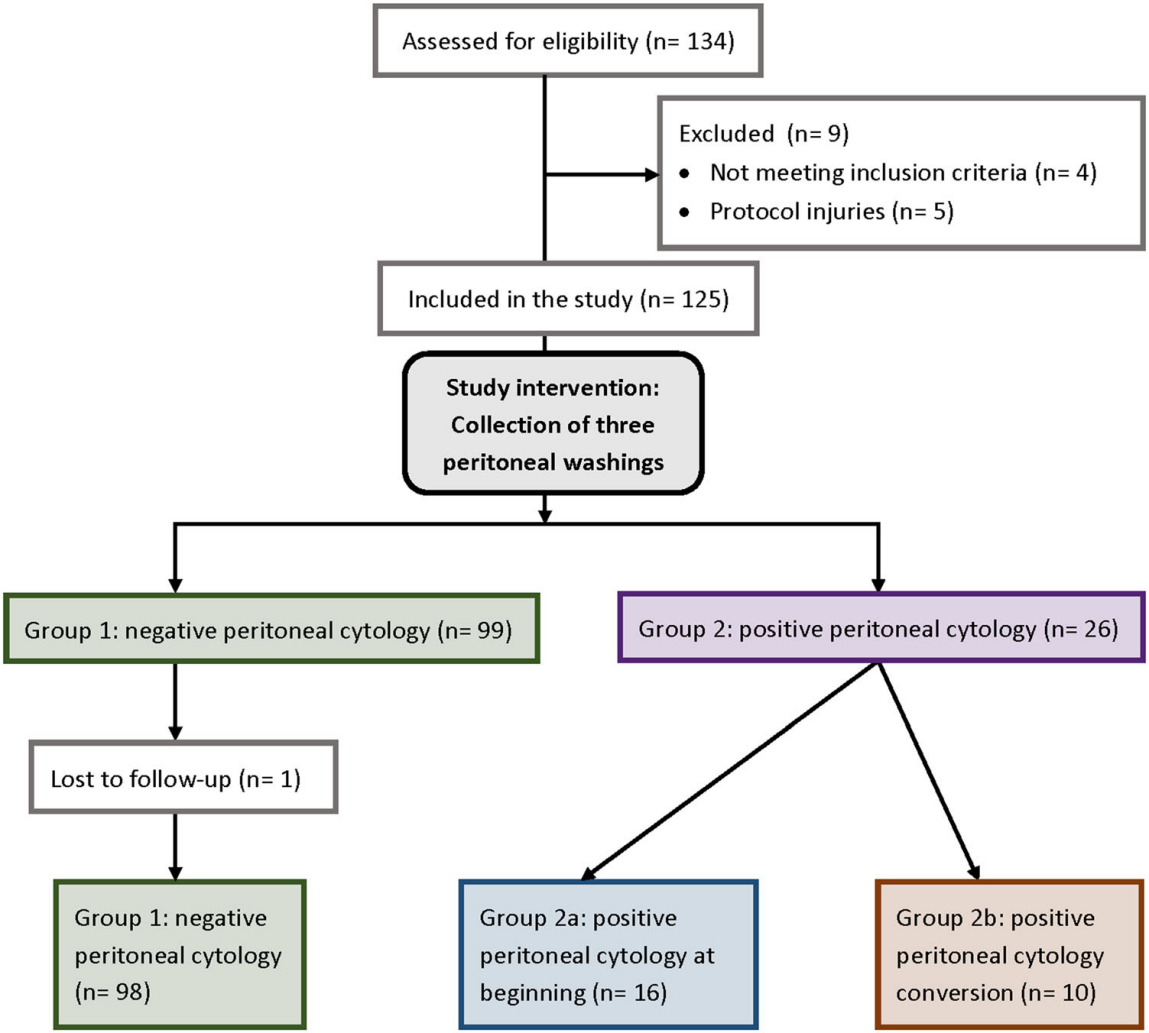
Consort diagram of the study

Data were collected at three national centers in Switzerland from October 2007 to December 2012. During the study period, 134 women underwent assessment for eligibility. Nine patients were excluded by reason of not fulfilling the inclusion criteria or owing to protocol injuries (nonadherence to the study intervention). The remaining 125 women met inclusion criteria and were enrolled in the present study. Of these, one patient with negative peritoneal cytology was lost to follow-up (Fig. 1). Mean age at surgery was 66.1 years, and mean BMI was 29.5 kg/m^2^. The majority of patients had low-grade tumors (75.0%) with endometrioid histology (87.9%) and stage I disease (78.2%). A total of 63.7% of patients underwent hysteroscopy prior to primary staging surgery, and 12.9% had a history of tubal sterilization. Table 1 presents the main baseline characteristics. All patients were operated with an intrauterine manipulating system: the RUMI® in 121 patients and the Hohl® in 3 patients. Uterine perforation occurred in two patients. Adjuvant treatment was performed in 64 (51.6%) patients: 36 patients were treated with adjuvant vaginal brachytherapy only, consisting of 28 with stage I, 6 with stage II, and 2 with stage III tumors. Of the two patients receiving vaginal brachytherapy and pelvic radiation, one had a stage III tumor and one a stage II tumor with high-grade histology. A total of 22 patients were treated with chemoradiation (15 with advanced stage disease and 7 with stage I disease and high-grade histology), and 5 patients with chemotherapy and vaginal brachytherapy (2 with advanced stage disease and 2 with stage I disease and high-grade histology).

**Table 1:** Patients demographics and surgical and histological baseline characteristics

	Total	Group 1: negative cytology	Group 2a: positive cytology at beginning	Group 2b: positive cytology conversion	*p-*value
Number of patients, *N* (%)	124 (100)	98 (79.0)	16 (12.9)	10 (8.1)	
Age at surgery, (years) mean* ±* SD	66.1 ± 10.0	66.3 ± 10.1	63.6 ± 9.4	68.0 ± 9.4	0.495
Postmenopausal, *N* (%)	113 (91.1)	89 (90.8)	15 (93.8)	9 (90.0)	0.921
Multiparous, *N* (%)	100 (80.6)	84 (85.7)	11 (68.8)	5 (50.0)	0.011
BMI, (kg/m^2^) mean ± SD	29.5 ± 8.1	30.1 ± 8.3	28.2 ± 7.5	26.2 ± 6.7	0.266
History of tubal sterilization, *N* (%)	16 (12.9)	13 (13.3)	2 (12.5)	1 (10.0)	0.957
Preoperative hysteroscopy, *N* (%)	79 (63.7)	64 (65.3)	9 (56.3)	6 (60.0)	0.759
Surgical lymph node staging performed, *N* (%)	95 (76.6)	76 (77.6)	14 (87.5)	5 (50.0)	0.080
*Grade, N (%)*					0.014
G1	45 (36.3)	32 (32.7)	5 (31.3)	8 (80.0)	
G2	48 (38.7)	43 (43.9)	4 (25.0)	1 (10.0)	
G3	31 (25.0)	23 (23.5)	7 (43.8)	1 (10.0)	
FIGO stage, *N* (%)					0.020
I	97 (78.2)	81 (82.7)	8 (50.0)	8 (80.0)	
II	7 (5.6)	6 (6.1)	1 (6.3)	0 (0.0)	
III	17 (13.7)	10 (10.2)	5 (31.3)	2 (20.0)	
IV	3 (2.4)	1 (1.0)	2 (12.5)	0 (0.0)	
Positive lymph node status, *N* (%)	15 (12.1)	8 (8.2)	7 (43.8)	0 (0.0)	< 0.001
Endometrioid histology, *N* (%)	109 (87.9)	86 (87.8)	13 (81.3)	10 (100)	0.317
LVSI positivity, *N* (%)	26 (21.0)	15 (15.3)	8 (50.0)	3 (30.0)	0.021
Adjuvant treatment, *N* (%)					0.145
None	58 (46.8)	49 (50.0)	3 (18.8)	6 (60.0)	
VBT	36 (29.0)	29 (29.6)	5 (31.3)	2 (20.0)	
VBT + EBRT	2 (1.6)	1 (1.0)	1 (6.3)	0 (0)	
Combined EBRT + CT	22 (17.7)	16 (16.3)	4 (25.0)	2 (20.0)	
VBT + CT	4 (3.2)	2 (2.0)	2 (12.5)	0 (0)	
Missing	2 (1.6)	1 (1.0)	1 (6.3)	0 (0)	
Follow-up, (months) mean (95% CI)	120 (116–125)	121 (117–126)	113 (96–130)	114 (100–129)	0.633
Recurrence, *N* (%)	19 (15.3)	9 (9.2)	4 (25.0)	6 (60.0)	< 0.001

*N* number*, SD* standard deviation, *CI* confidence interval, *BMI* body mass index*, FIGO* Federation International de Gynecologie et Obstetrique*, LVSI* lymphovascular space invasion, *VBT* vaginal brachytherapy, *EBRT* external beam radiotherapy, *CT* chemotherapy

### Peritoneal Cytology

In total, 43 peritoneal cytologies showed evidence of malignant cells, including 16 premanipulator, 11 postmanipulator, and 16 posthysterectomy washings. 33 of the 43 positive peritoneal cytologies showed indeterminate results on conventional hematoxylin–eosin staining and were diagnosed on the basis of immunohistochemistry. A total of 98 (79.0%) patients presented with a negative peritoneal cytology (group 1) while 26 (21.0%) patients had at least one positive peritoneal washing (group 2). There was a significant correlation between positive peritoneal cytology and the following histopathological characteristics: advanced FIGO stage (*p* = 0.025), lymph node involvement (*p* = 0.017), LVSI (*p* = 0.015), and myometrial invasion (*p* = 0.030). No significant association was seen between peritoneal cytology and uterine perforation (*p* = 0.623), type of manipulator (*p* = 0.497), preoperative workup with hysteroscopy (*p* = 0.310), grading (*p* = 0.056), and histological subtype (*p* = 0.554). In group 2, 16 patients had positive premanipulator cytology (group 2a) and 10 patients had initially negative peritoneal washings, converting either after insertion of the manipulator (two patients) or at the end of the surgical procedure (8 patients), forming group 2b. Patients in group 2b all had endometrioid histologies, and the majority (80%) had stage I disease (Table 1). Eight out of these ten positive converted cytologies showed evidence of malignant cells on the basis of immunohistochemistry only.

### Oncological Outcome

**Fig. 2:**
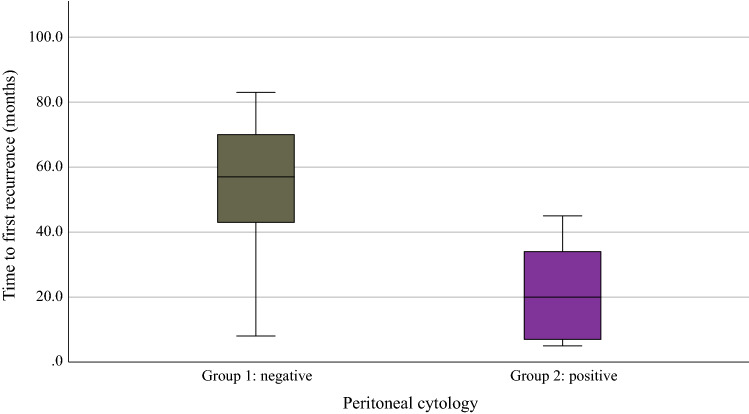
Association between peritoneal cytology and mean time to first recurrence (log-rank, *p* = 0.004)

Mean follow-up was 120.7 [95% confidence interval (CI) 116.2–125.2] months, and a total of 19 (15.3%) patients experienced at least one recurrence. Recurrence rate differed significantly among the distinct study groups: in group 1, nine (9.2%) patients experienced recurrence, while it was four (25%) and six (60%) patients in group 2a and 2b, respectively (Table 1, *p* < 0.001). Mean time to first recurrence was 34.8 (95% CI 23.6–46.1) months for all recurrent patients. Patients with negative peritoneal washings had a significantly longer time to first recurrence of 50.4 (95% CI 33.9–67.0) months compared to 20.8 (95% CI 11.8–29.8) months in patients with positive cytologies (log-rank, *p* = 0.004; Fig. 2), including 21.0 (95% CI 10.8–31.2) months in group 2a and 20.7 (95% CI 6.5–34.9) months in group 2b. The majority (70.0%) of recurrences in group 2 occurred during the first two years after primary treatment, whereas it was only 22.2% of the recurrences in group 1 (*p* = 0.051). Four patients experienced recurrence more than five years after primary treatment; all of these had negative peritoneal cytology (*p* = 0.033).

**Fig. 3:**
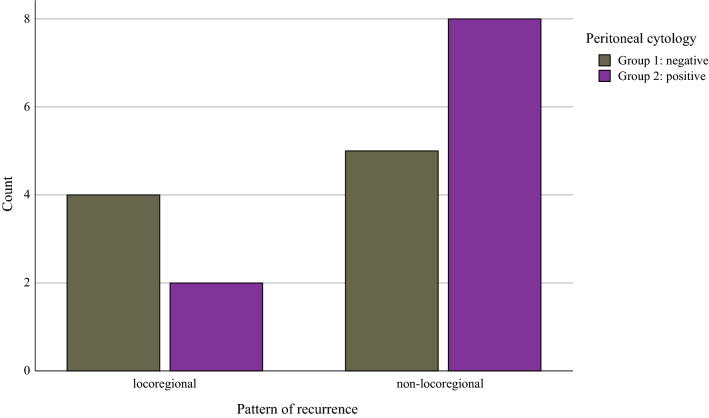
Association of pattern of recurrence and peritoneal cytology (*χ*^2^, *p* = 0.259)

The 19 recurrences consisted of 5 locoregional, 6 abdominal, and 8 distant recurrences. In group 1, localization of recurrences was distributed as follows: four (44.4%) locoregional, two (22.2%) abdominal, and three (33.3%) distant. Group 2 comprises two (20%) locoregional, three (30%) abdominal, and five (50%) distant recurrences, respectively (*p* = 0.517). Patients with positive cytology showed numerically more nonlocoregional recurrences (80.0%) compared with patients with negative cytology (55.6%), although this observation was not statistically significant (*p* = 0.259; Fig. 3).

**Fig. 4:**
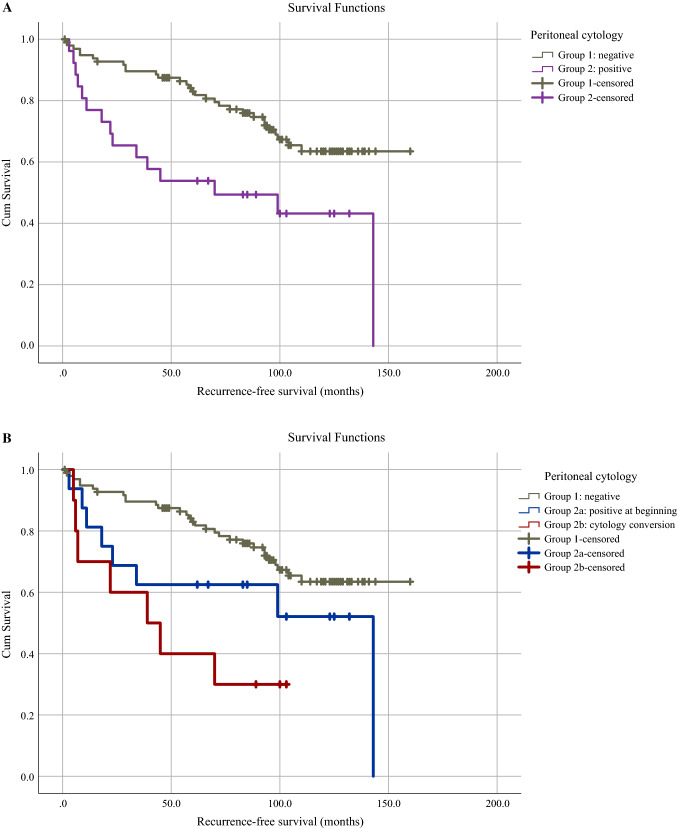

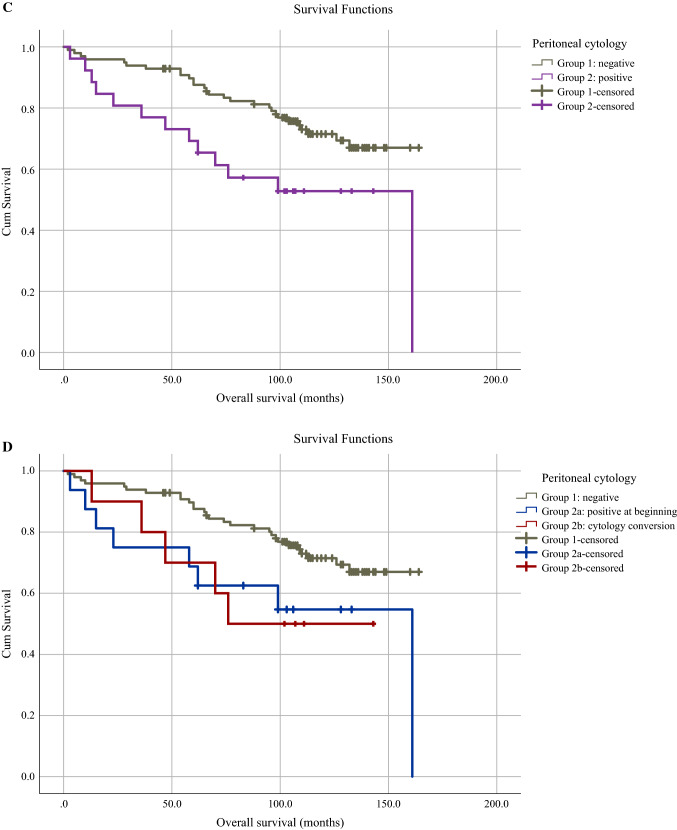
Kaplan–Meier survival curves according to peritoneal cytology. **A** recurrence-free survival in patients with negative (group 1) and positive (group 2) peritoneal cytology (log-rank, *p* = 0.003); **B** recurrence-free survival in patients with negative peritoneal cytology (group 1), positive peritoneal cytology at the beginning of the procedure (group 2a) and positive cytology conversion (group 2b) (log-rank, *p* = 0.002); **C** overall survival in patients with negative (group 1) and positive (group 2) peritoneal cytology (log-rank, *p* = 0.016); **D** overall survival in patients with negative peritoneal cytology (group 1), positive peritoneal cytology at the beginning of the procedure (group 2a) and positive peritoneal cytology conversion (group 2b) (log-rank, *p* = 0.053)

Forty-one (33.1%) patients died during follow-up, involving 28 deaths (28.6%) in group 1, 8 (50.0%) in group 2a, and 5 (50%) in group 2b. Mean recurrence-free survival was 112.5 (95% CI 101.0–124.0) months for the whole study population, with the best survival for patients with negative cytology, followed by patients from group 2a and 2b with 123.8 (95% CI 112.9–134.8), 90.9 (95% CI 59.2–122.6), and 50.3 (95% CI 25.8–74.8) months, respectively (log-rank, *p* = 0.002). Similar associations were seen in overall survival, amounting to 127.7 (95% CI 118.3–137.1) months for the whole study cohort and 134.6 (95% CI 125.0–144.3), 106.5 (95% CI 72.6–140.3), and 95.7 (95% CI 64.7–126.7) months for groups 1, 2a, and 2b, respectively (log-rank, *p* = 0.053). The corresponding Kaplan–Meier curves are shown in Fig. 4. On univariable analysis, the following prognostic factors were significantly associated with a higher risk of recurrence and death: positive peritoneal cytology, FIGO stage >I, and deep myometrial invasion. Lymph node involvement and age at surgery showed significant higher risk of death only. On multivariable analysis, positive peritoneal cytology remained a significant independent predictor of risk of recurrence [hazard ratio (HR) 4.15, 95% CI 1.501–11.482, *p* = 0.006] and death (HR 2.92, 95% CI 1.218–6.980, *p* = 0.016) and age at surgery a predictor of death (HR 1.08, 95% CI 1.040–1.131, *p* = < 0.001) (Table 2).

**Table 2:** Cox regression for univariable and multivariable analysis for risk of recurrence (a) and risk of death (b) according to clinicopathological features

Clinicopathological factor	Univariable analysis			Multivariable analysis		
	HR	95% CI	*p*-value	HR	95% CI	*p*-value
*(a) Risk of recurrence*						
Prior hysteroscopy			0.116	–	–	–
No (ref.)	1.0	Reference				
Yes	2.42	0.804–7.311				
Age at surgery (years)	1.04	0.989–1.090	0.127	–	–	–
Type of manipulator			0.641	–	–	–
RUMI (ref.)	1.0	Reference				
Hohl	0.05	0.000–17299				
Intraoperative perforation			0.678	–	–	–
No (ref.)	1.0	Reference				
Yes	0.05	0.000–77838				
Peritoneal cytology			**< 0.001**			**0.006**
negative (ref.)	1.0	Reference		1.0	Reference	
positive	5.44	2.205–13.42		4.15	1.501–11.482	
Histologic subtype			0.517	–	–	–
Endometrioid (ref.)	1.0	Reference				
Non-endometrioid	1.50	0.438–5.160				
LVSI			0.869	–	–	–
No (ref.)	1.0	Reference				
Yes	0.91	0.293–2.821				
Grading			0.276	–	–	–
Low grade (G1, G2) (ref.)	1.0	Reference				
High grade (G3)	1.68	0.660–4.273				
Myometrial invasion			**0.043**			0.195
< 50% (ref.)	1.0	Reference		1.0	Reference	
> 50%	2.77	1.034–7.398		1.97	0.707–5.484	
FIGO stage			**0.017**			0.594
I (ref.)	1.0	Reference		1.0	Reference	
> I	3.05	1.224–7.599		1.34	0.451–4.016	
Lymph node status			0.124	–	–	–
Negative (ref.)	1.0	Reference				
Positive	2.46	0.782–7.743				
History of tubal sterilization			0.751	–	–	–
No (ref.)	1.0	Reference				
Yes	0.79	0.182–3.417				
Adjuvant treatment			0.113	–	–	–
None (ref.)	1.0	Reference				
RT	1.7	0.537–5.179				
RT and CT	3.3	0.901–9.695				

Clinicopathological factor	Univariable analysis			Multivariable analysis		
	HR	95% CI	*p*-value	HR	95% CI	*p*-value
*(b) Risk of death*						
Prior hysteroscopy			0.353	–	–	–
No (ref.)	1.0	Reference				
Yes	1.37	0.707–2.644				
Age at surgery (years)	1.08	1.039–1.114	**< 0.001**	1.08	1.040–1.131	**< 0.001**
Type of manipulator			0.739	–	–	–
RUMI (ref.)	1.0	Reference				
Hohl	1.30	0.298–6.037				
Intraoperative perforation			0.846	–	–	–
No (ref.)	1.0	Reference				
Yes	1.22	0.167–8.873				
Peritoneal cytology			**0.019**			**0.016**
Negative (ref.)	1.0	Reference		1.0	Reference	
Positive	2.23	1.144–4.340		2.92	1.218–6.980	
Histologic subtype			0.128	–	–	–
Endometrioid (ref.)	1.0	Reference				
Non-endometrioid	1.89	0.834–4.262				
LVSI			0.814	–	–	–
No (ref.)	1.0	Reference				
Yes	1.09	0.526–2.264				
Grading			0.224	–	–	–
Low grade (G1, G2) (ref.)	1.0	Reference				
High grade (G3)	1.51	0.777–2.930				
Myometrial invasion			**0.007**			0.274
< 50% (ref.)	1.0	Reference		1.0	Reference	
> 50%	2.38	1.262–4.477		1.51	0.723–3.138	
FIGO stage			**0.012**			0.850
I (ref.)	1.0	Reference		1.0	Reference	
> I	2.30	1.198–4.403		1.13	0.316–4.054	
Lymph node status			**0.016**			0.372
Negative (ref.)	1.0	Reference		1.0	Reference	
Positive	2.54	1.186–5.415		1.93	0.457–8.123	
History of tubal sterilization			0.804	–	–	–
No (ref.)	1.0	Reference				
Yes	1.13	0.440–2.881				
Adjuvant treatment			0.138	–	–	–
None (ref.)	1.0	Reference				
RT	1.7	0.804–3.468				
RT and CT	2.1	0.979–4.638				

A statistically significant *p*-value below 0.05 was marked bold in the table

*HR* hazard ratio, *CI* confidence interval, *FIGO* Federation International de Gynecologie et Obstetrique, *LSVI* lymphovascular space invasion, *RT* radiotherapy, *CT* chemotherapy

## Discussion

The use of intrauterine manipulators during minimal invasive surgery for endometrial cancer remains controversial. In our multicenter, prospective, clinical trial, we evaluated the impact of intrauterine manipulation on peritoneal cytology and oncological outcome. Our results demonstrated positive peritoneal cytology conversion in 8.1% of patients with endometrial cancer undergoing minimally invasive surgery with intrauterine manipulation. Furthermore, there was a significant correlation of recurrence rate with peritoneal cytology, and patients with converted peritoneal cytology showed the worst oncological outcomes.

In our cohort, 12.9% of patients presented with positive peritoneal cytology at the beginning of surgery. This is consistent with current literature, revealing positive peritoneal cytology rates in endometrial cancer ranging from 5% to 20%.^26,29,30^ In total, 8.1% of our study patients had initially negative peritoneal washings but converted during procedure. Previously published articles have shown a similar trend toward a higher rate of positive peritoneal cytology conversion after the use of intrauterine manipulators. However, for the most part, the results did not meet statistical significance,^16,17,40^ mainly owing to small sample sizes. Furthermore, the majority of the studies investigated only one additional peritoneal washing after insertion of the uterine manipulator.^15,16,41^ In our population, 80% of the converted cytologies were found only at the end of the surgery, suggesting that the issue is not mainly the insertion of the manipulator but rather the manipulation during the whole procedure. The only further study investigating three sets of peritoneal washings to date, by Lim et al., found two converting positive cytologies in 46 patients, one postmanipulator and one posthysterectomy—corresponding to a conversion rate of 4.3%.^40^ Since our cohort showed no association between uterine perforation and peritoneal cytology conversion, we assume rather a microscopic dissemination pathway on the basis of increased intrauterine pressure facilitating the ability of tumor cells to exceed the myometrial barrier and to spread outside the uterine cavity.^20,21^ The possible spread via the fallopian tubes was minimized in our study by performing bipolar coagulation of the fallopian tubes prior to the insertion of the manipulator.

Our results demonstrated significantly higher recurrence rates in patients with positive peritoneal cytology and highest recurrence rates in group 2b (Table 1) despite highly favorable histological features. Accordingly, recurrence-free and overall survival were best in patients with negative peritoneal cytology and worst in group 2b (Fig. 4). While the association of positive peritoneal cytology with decreased survival has been shown by multiple studies,^26–30^ no study has explored the impact of cytology conversion on oncological outcome in endometrial cancer to date. On the other hand, several studies investigating the impact of intrauterine manipulators on oncological outcomes revealed no significant association,^15–18,42,43^ except for the largest study by Padilla et al.^20^ This might be due to the generally low risk of recurrence in endometrial cancer, making larger sample sizes necessary to detect significant differences. Nevertheless, in some of the above-mentioned studies, there was a trend toward worse oncological outcomes with the use of intrauterine manipulators, although not statistically significant.^15-17^

Mean time to first recurrence was 34.8 months in our study. Time to first recurrence was significantly shorter in patients with positive cytologies (Fig. 2), in line with the assumption that a short time to recurrence in endometrial cancer does not necessarily indicate treatment resistance but may be related to the persistence of microscopic disease after primary treatment.^44^

A trend toward more nonlocoregional recurrences related to the use of intrauterine manipulators was described both in our cohort (Fig. 3) and the study of Padilla et al.^20^ We interpret this in the context of the pressure effect of the manipulator on the tumor microenvironment: the increased pressure inside the uterine cavity potentially helps to spread tumor cells into the blood circulation intraoperatively.

### Clinical Relevance of Our Findings

Our study supplies crucial knowledge for understanding the impact of the use of intrauterine manipulators on oncological outcomes in patients with endometrial cancer. The results of this study provide further evidence to fill the remaining gaps between uterine manipulation, peritoneal cytology, and recurrence in endometrial cancer. Our findings suggest that the use of intrauterine manipulators may lead to positive peritoneal cytology conversion, which in turn enhances recurrence rate. This correlation is different from hysteroscopy, where current literature reports a higher frequency of positive peritoneal cytology but without impact on oncological outcome.^32–36^ Potentially, hysteroscopy results in a rather passive rinsing out of tumor cells into the abdominal cavity while intrauterine manipulation leads to trauma and inflammation possibly triggering disease recurrence by shedding of tumor cells into the blood and lymphatic circulation.^45^ In addition, in hysteroscopy, there is a lack of prospective data showing the relationship between hysteroscopy, peritoneal cytology, and oncological outcome.

Minimal invasive surgery certainly remains the standard of care in endometrial cancer treatment after the results of prospective randomized trials proving its oncological safety.^5,6^ Unfortunately, these trials did not report on the use of uterine manipulators. Since there are no data proving that the use of uterine devices reduces surgical complications,^13^ and current evidence supports that there is a safe possibility to perform hysterectomy without the use of intrauterine manipulators,^46,47^ we should consider abandoning intrauterine manipulators in surgery for endometrial cancer. Particularly, after the unexpected results of the Laparoscopic Approach to Cervical Cancer (LACC) trial where tumor spill secondary to the use of an uterine manipulator was named as one of the possible contributors to the worse oncological outcome in patients with cervical cancer after minimally invasive surgery.^21,48^ However, larger prospective clinical trials, including a control group operated without intrauterine manipulator, are needed to confirm our results.

### Strengths and Weaknesses

In our opinion, the major strengths of this study include its prospective and multicenter design as well as the long-term follow-up. To the best of our knowledge, this is the first study exploring the impact of cytology conversion on oncological outcome in endometrial cancer to date. One of its most interesting aspects is the inclusion of a third cytology, collected at the end of surgery, thereby not only investigating the effect of the insertion of the manipulator but also the impact of the intrauterine manipulation during the whole procedure. The most important shortcoming of the current study is the lack of a control group operated without intrauterine manipulator. Furthermore, statistical hypothesis and sample size were not calculated for oncological outcomes. Subsequently, our results cannot establish the responsibility of the intrauterine manipulator for the cytology conversion and the worse oncological outcome.

## Conclusions

This multicenter prospective trial showed a rate of 8.1% of peritoneal cytology conversion in patients with endometrial cancer undergoing minimal invasive surgical staging procedure with the use of an intrauterine manipulator. Our results revealed a strong correlation of recurrence rate with peritoneal cytology, and patients with converted peritoneal cytology presented with the worst oncological outcomes.
